# Thrombocytopenia induced by dabigatran: two case reports

**DOI:** 10.1186/s12883-017-0900-8

**Published:** 2017-06-29

**Authors:** Hyun Goo Kang, Seung Jae Lee, Ji Yeon Chung, Jin Sung Cheong

**Affiliations:** 1 Department of Neurology, Chosun University School of Medicine, Gwangju, 61453 Republic of Korea; 2 Research Center for Bioactive Materials and Department of Chemistry, Chonbuk National University, Jeonju, 54896 Republic of Korea; 3 Department of Neurology, Wonkwang University School of Medicine and Hospital, Iksan, 54538 Jeonbuk Republic of Korea

**Keywords:** Dabigatran, Factor Xa inhibitor, Thrombin inhibitor, Thrombocytopenia

## Abstract

**Background:**

Vitamin K inhibitors (e.g. warfarin) and indirect thrombin inhibitors (e.g. heparin) are widely used to prevent thromboembolic disorders (e.g. myocardial infarction, venous thromboembolism, and stroke). These agents have been mainstays of anticoagulation for people older than 60 years. However, their administration is associated with a risk of bleeding and requires careful monitoring of patients. Novel oral anticoagulants (NOACs), such as dabigatran, are significantly safer in preventing thromboembolism than warfarin and heparin (sporadically causes thrombocytopenia) and are more specific for their target protein, thrombin. The major advantage of dabigatran, a direct thrombin inhibitor, is that it reversibly inhibits both free and clot-bound thrombin by tight binding affinity and the predictable pharmacodynamic effect. A few studies, however, reported that dabigatran can cause thrombocytopenia, although the underlying mechanism remains unclear. Thus, an antidote for dabigatran was developed to prevent thrombocytopenia.

**Case presentation:**

In this report, we discuss two cases of thrombocytopenia and purpura after dabigatran treatment. A 73-year-old man showed hemorrhagic necrotic skin lesions on his neck and right hand. He was administered dabigatran (220 mg/day) for cerebral infarction for three days and his platelet count decreased abruptly (6000/μL). This suggested that dabigatran had caused thrombocytopenia and purpura; therefore, dabigatran administration was discontinued. The results of a blood test, performed 14 days after stopping dabigatran treatment, showed that the platelet count had recovered to the normal range of more than 150,000/μL. A 75-year-old woman had taken warfarin continuously for 8 years. However, she had a new cerebral infarction. Therefore, warfarin treatment was replaced with dabigatran (300 mg/day). Her platelet count decreased (41,000/μL) significantly and dabigatran treatment was discontinued. The blood test results show that platelet counts gradually recovered to the normal range.

**Conclusions:**

Dabigatran application may cause bleeding; therefore, careful monitoring during dabigatran treatment is required to prevent thrombocytopenia. An explanation is that the interaction of dabigatran with thrombin, because of its strong binding affinity, may cause the observed thrombocytopenia.

## Background

Thromboembolic disorders are a major cause of myocardial infarction, venous thromboembolism, and stroke secondary to hypercoagulation [1, 2]. Warfarin, a vitamin K antagonist, and heparin, an indirect thrombin inhibitor, were used for many years to achieve anticoagulation, although, these treatments can occasionally cause bleeding given their narrow therapeutic window and non-specific interactions [3, 4]. Novel oral anticoagulants (NOACs) work rapidly, with minimal interactions with food and/or medication and allow the prediction of anticoagulation without intense monitoring. Dabigatran, a reversible and a direct thrombin inhibitor, was demonstrated to have a better safety profile than heparin, which sporadically causes thrombocytopenia [5]. Some studies, however, reported that dabigatran can cause thrombocytopenia, although the underlying mechanism remains unclear [6]. Herein, we report two cases of thromboembolism and purpura after dabigatran treatment and discuss the clinical features and possible underlying mechanisms.

## Case presentation

**Fig. 1:**
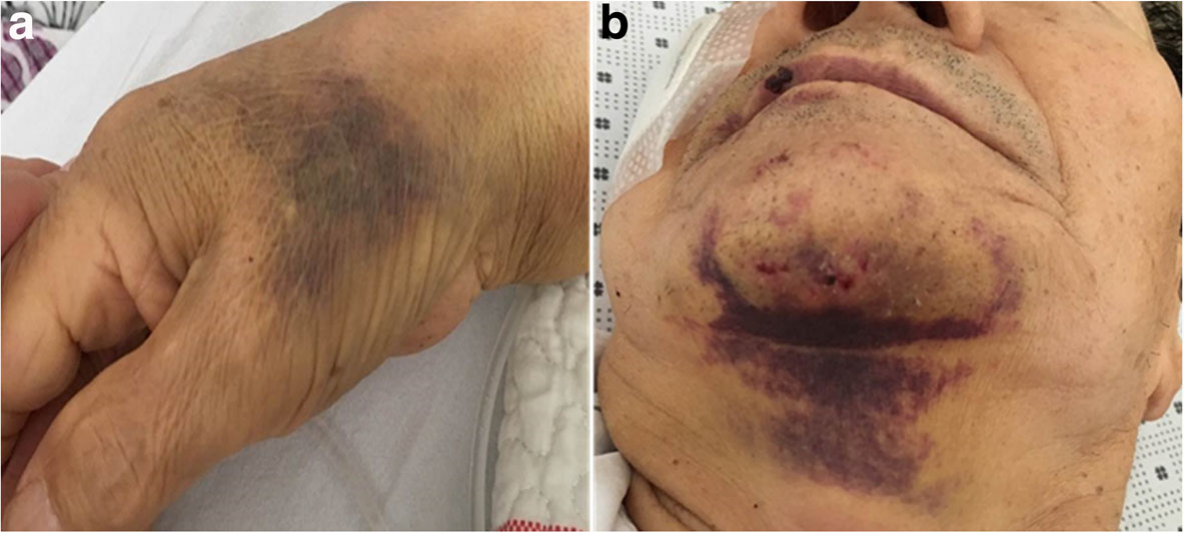
Bruising and multiple petechiae on the patient’s right hand (**a**) and chin (**b**) in patient 1 after 14 days treatment of dabigatran

**Fig. 2:**
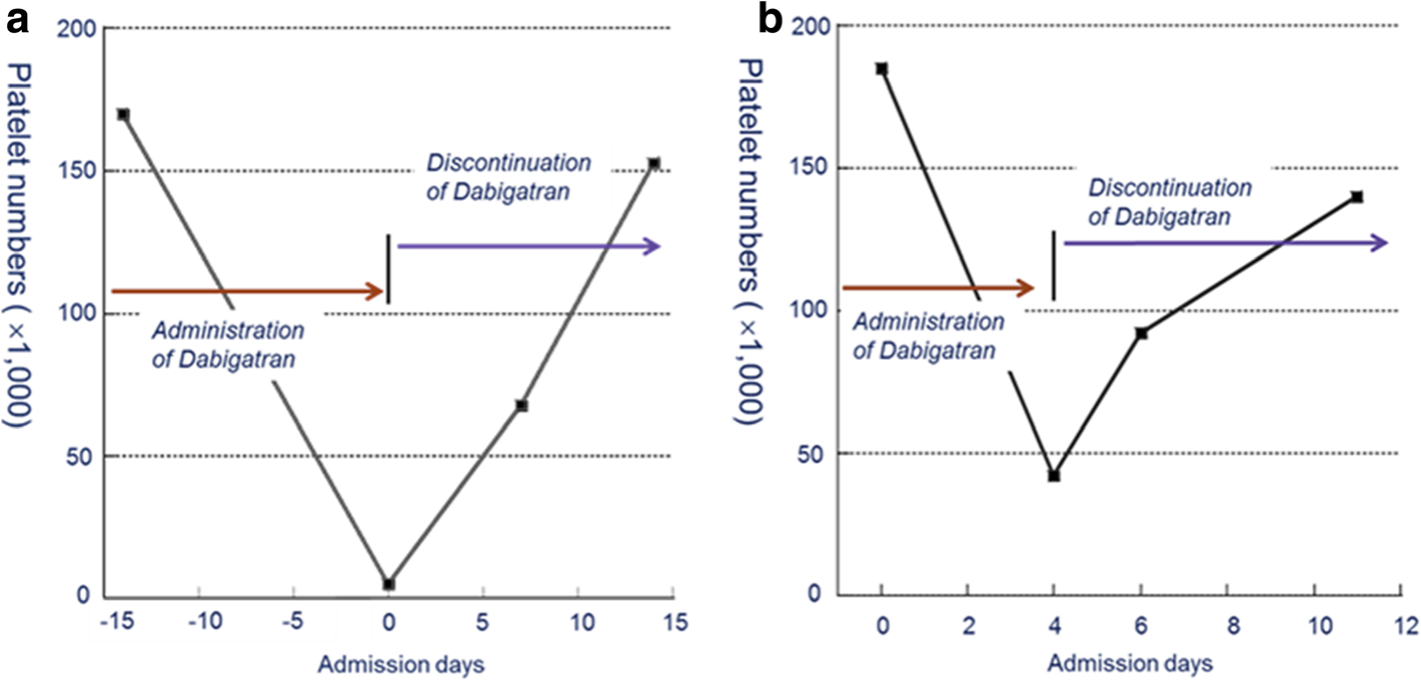
Course of thrombocytopenia caused by dabigatran in patient 1 and 2. Platelet counts were gradually recovered after discontinuation of dabigatran

A 73-year-old man was referred to our neurology clinic because of bruises on the back of the hands and chin with multiple petechiae that appeared 3 days before admission. Hemorrhagic necrotic skin lesions were detected on his neck and right hand by physical examination (Fig. 1a-b). For the past 5 years, he had undergone hypertension treatment and gout treatment for the last year. Cerebral infarction occurred on his left frontal lobe two weeks prior to admission, and permanent atrial fibrillation was identified by 24-h Holter test. Dabigatran (220 mg/day) was prescribed when he was hospitalized. He did not present any abnormal vital signs or neurological disorders. On day 15 after starting dabigatran treatment the blood tests showed that white blood cell, red blood cell, hemoglobin, hematocrit, and platelets counts were 5800/μL, 2890/μL, 9.3 g/dl, 27.8%, and 6000/μL, respectively, indicating that the number of platelets had decreased dramatically (Fig. 2a **)**. A peripheral blood smear test showed severe thrombocytopenia without anisocytosis. A blood test performed 5 months earlier at the clinic demonstrated that the platelet count (164,000/μL) and antiplatelet antibody were within normal physiological range. Prothrombin time and activated partial thromboplastin time results were normal. There was no evidence of pseudothrombocytopenia. We suspected that dabigatran caused thrombocytopenia and purpura, and thus discontinued the dabigatran treatment. After 14 days the blood test indicated that the platelet count was again within the normal range (152,000/μL) (Fig. 2a).

A 75-year-old woman had taken warfarin continuously for 8 years with stable International Normal Ratio (INR) since she was diagnosed with cerebral infarction and hypertension. This patient was referred to our clinic because of new development of acute aphasia and right hemiplegia. A new cerebral infarction was monitored in the left middle cerebral artery territory, which was confirmed by magnetic resonance imaging. At hospitalization, her platelet count (176,000/μL) and antiplatelet antibody were normal. The patient did not show any critical petechiae or abnormal vital signs. A transthoracic echocardiography was conducted after admission with no abnormal findings except for permanent atrial fibrillation. Therefore, we changed treatment drug from warfarin to dabigatran (300 mg/day), although she was within the therapeutic warfarin range 65.7% at that time [3]. On day 4 of dabigatran treatment, the blood tests showed a marked decrease in platelets (41,000/μL, Fig. 2b), although other results including white blood cell count (6100/μL), red blood cell count (3190/μL), hemoglobin levels (10.4 g/dL), and hematocrit levels (29.3%), were within the physiological range. Prothrombin time and activated partial thromboplastin time were optimal. Thus, dabigatran can cause thrombocytopenia and therefore dabigatran treatment was discontinued. Blood tests conducted 11 days after dabigatran discontinuation showed a platelet count of 134,000/μL, indicating a gradual recovery. In this case, warfarin treatment showed good clinical results and was effective for this patient.

## Discussion and conclusions

**Fig. 3:**
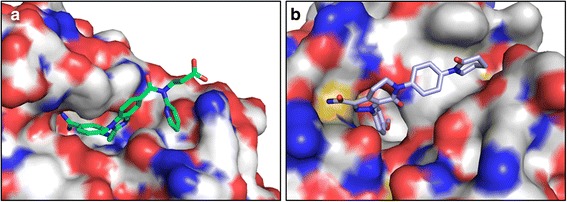
Active sites of (**a**) Dabigatran-thrombin (PDB accession: 4YHI) and (**b**) Apixaban-factor Xa (PDB accession: 2P16). These inhibitors generate specific interactions to their target enzymes to prevent anti-coagulation. The hydrophobic and hydrophobic pharmacophores interact to their target enzymes. *Blue*, *red*, and *gray* represent the nitrogen, oxygen, and carbon atoms

The recently introduced new oral anticoagulant (NOACs), dabigatran etexilate, is a potent non-peptidic thrombin inhibitor that reversibly binds to the active site of thrombin to prevent the formation of fibrin clots [7–10]. The clinical recommendations for dabigatran use and therapeutic safety based on its dosing regimen were established in preliminary pharmacokinetics and pharmacodynamics studies [5, 8, 11–13]. Despite the development of NOACs, such as dabigatran, the risk of bleeding remains a major concern during clinical treatment. The case report by Deidda et al. indicated that thrombocytopenia is rarely caused by dabigatran, but the possibility of a life-threatening complication was suggested [6]. Given the short half-life of the interaction between dabigatran and thrombin (Fig. 3a), combination treatment with a vitamin K inhibitor and factor Xa inhibitor, such as apixaban, (Fig. 3b) was prescribed in critical situations [12–14]. Idarucizumab was developed as an antidote for dabigatran to avoid hemorrhage via the reversal of anticoagulant effects. This antidote has a 300-fold stronger binding affinity to thrombin than dabigatran, and it can avoid critical bleeding secondary to anticoagulation [2, 10, 15, 16]. Recent reports also proposed a heparin-induced thrombocytopenia (HIT) mechanism which demonstrates a relationship of pathogenic antibodies and the complex generation between platelet factor 4 (PF4) and heparin [17]. This complex triggers a signaling cascade, which induces severe complications. So, further research is required to understand the interaction between NOACs and their receptors.

We postulate that dabigatran can lead to hemorrhagic events because of its strong binding affinity, although it selectively and specifically interacts with thrombin. The dissociation constant (K_d_) of dabigatran is a reflection of its very strong binding affinity, with a sub-nanomolar range [7, 12, 14]. Based on the X-ray crystallography analysis, the structure of dabigatran generates specific interaction with the surface of thrombin through hydrophobic and hydrophilic bonds. Computational studies of the dabigatran-thrombin complex showed hydrogen bonds with nitrogen and oxygen atoms (Fig. 3a). Additionally, the hydrophobic cores, such as benzimidazole and pyridine, are an important scaffold acting as a pharmacophore for its anticoagulant activity [2, 11]. The carboxylate group in dabigatran can generate hydrogen bonds with free water and other molecules that help to release dabigatran. The reversal of dabigatran activity is based on the pharmacokinetic and physical properties of these carboxylic acids. The amidino groups generate hydrogen bonds with the aspartate189 (Asp189) residue of thrombin. Based on the present cases, we conclude that dabigatran can possibly cause thrombocytopenia and careful monitoring is required during dabigatran treatment to avoid possible side-effects such as hemorrhagic evets.

## Abbreviations

INR: International normalised ratio
NOACs: Novel oral anticoagulants

## References

[1] Illanes S, Zhou W, Schwarting S, Heiland S, Veltkamp R (2011). Comparative effectiveness of hemostatic therapy in experimental warfarin-associated intracerebral hemorrhage. Stroke.

[2] Patel NR, Patel DV, Murumkar PR, Yadav MR (2016). Contemporary developments in the discovery of selective factor Xa inhibitors: a review. Eur J Med Chem.

[3] Hart RG, Pearce LA, Aguilar MI (2007). Meta-analysis: antithrombotic therapy to prevent stroke in patients who have nonvalvular atrial fibrillation. Ann Intern Med.

[4] Riva N, Lip GYH (2012). A new era for anticoagulation in atrial fibrillation which anticoagulant should we choose for long-term prevention of thromboembolic complications in patients with atrial fibrillation?. Pol Arch Med Wewn.

[5] Skelley JW, Kyle JA, Roberts RA (2016). Novel oral anticoagulants for heparin-induced thrombocytopenia. J Thromb Thrombolys..

[6] Deidda A, Rapallo M, Sofia MD, MNeloni L, Lampus SM (2015). Thrombocytopenia possibly induced by dabigatran: a case report. Aust J Pharm.

[7] Eisert WG, Hauel N, Stangier J, Wienen W, Clemens A, van Ryn J (2010). Dabigatran: an oral novel potent reversible nonpeptide inhibitor of thrombin. Arterioscl Throm Vas.

[8] Pollack CV, Reilly PA, Bernstein R, Dubiel R, Eikelboom J, Glund S (2015). Design and rationale for RE-VERSE AD: a phase 3 study of idarucizumab, a specific reversal agent for dabigatran. Thromb Haemost.

[9] Pragst I, Zeitler SH, Doerr B, Kaspereit FJ, Herzog E, Dickneite G (2012). Reversal of dabigatran anticoagulation by prothrombin complex concentrate (Beriplex P/N) in a rabbit model. J Thromb Haemost.

[10] van Ryn J, Goss A, Hauel N, Wienen W, Priepke H, Nar H, Clemens A (2013). The discovery of dabigatran etexilate. Front Pharmacol.

[11] Ren WX, Ren YJ, Wang S (2016). Design, synthesis, anticoagulant activity evaluation and molecular docking studies of a class of N-ethyl dabigatran derivatives. Eur J Med Chem.

[12] Schiele F, van Ryn J, Canada K, Newsome C, Sepulveda E, Park J (2013). A specific antidote for dabigatran: functional and structural characterization. Blood.

[13] Wong PC, Pinto DJP, Zhang DL (2011). Preclinical discovery of apixaban, a direct and orally bioavailable factor Xa inhibitor. J Thromb Thrombolys.

[14] Schiele F, van Ryn J, Litzenburger T, Ritter M, Seeliger D, Nar H (2015). Structure-guided residence time optimization of a dabigatran reversal agent. MAbs.

[15] Pollack CV, Reilly PA, Eikelboom J (2015). Bleeding Antidote against Dabigatran: Idarucizumab. Aktuel Neurol.

[16] Zhou W, Schwarting S, Illanes S, Liesz A, Middelhoff M, Zorn M (2011). Hemostatic therapy in experimental intracerebral hemorrhage associated with the direct thrombin inhibitor dabigatran. Stroke.

[17] Welsby IJ, Krakow EF, Heit JA, Wiliams EC, Arepally GM, Bar-Yosef S, et al. The Association of Anti-Platelet Factor 4/Heparin Antibodies With Early and Delayted Thromboembolism After Cardiac Surgery. J Thromb Haemost 2017, 15:57–65s. 10.1111/jth.13533 PMC5280211 27714919

